# Acetohydrazide

**DOI:** 10.1107/S1600536809042469

**Published:** 2009-10-23

**Authors:** Bao-Han Zhou

**Affiliations:** a Chemical and Environmental Engineering Department, Hu Bei University of Technology, Wuhan 430068, People’s Republic of China

## Abstract

In the title compound, C_2_H_6_N_2_O, a hydrazine derivative, the asymmetric unit contains two mol­ecules with similar geom­etries. The crystal structure is stabilized by inter­molecular N—H⋯O hydrogen bonds.

## Related literature

For general background to hydrazine and its derivatives, see: Gagnon *et al.* (1951 ▶); Hermanson (1996 ▶); Lumley-Woodyear *et al.* (1996 ▶); Raddatz *et al.* (2002 ▶).
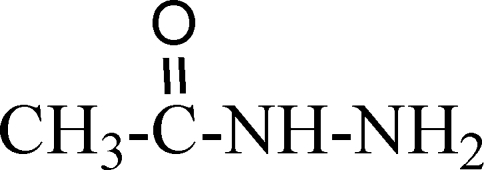


## Experimental

### 

#### Crystal data

C_2_H_6_N_2_O
*M* *_r_* = 74.09
Monoclinic, 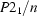

*a* = 9.5636 (7) Å
*b* = 8.7642 (6) Å
*c* = 10.4282 (7) Å
β = 110.886 (1)°
*V* = 816.63 (10) Å^3^
*Z* = 8
Mo *K*α radiation
μ = 0.10 mm^−1^
*T* = 298 K
0.20 × 0.15 × 0.10 mm

#### Data collection

Bruker SMART 4K CCD diffractometer
Absorption correction: multi-scan (*SADABS*; Bruker, 2001 ▶) *T*_min_ = 0.971, *T*_max_ = 0.990
4189 measured reflections
1762 independent reflections
1604 reflections with *I* > 2σ(*I*)
*R*_int_ = 0.097

#### Refinement

*R*[*F* ^2^ > 2σ(*F* ^2^)] = 0.056
*wR*(*F* ^2^) = 0.151
*S* = 1.15
1762 reflections
112 parameters
6 restraints
H atoms treated by a mixture of independent and constrained refinement
Δρ_max_ = 0.19 e Å^−3^
Δρ_min_ = −0.19 e Å^−3^

### 

Data collection: *SMART* (Bruker, 2001 ▶); cell refinement: *SAINT-Plus* (Bruker, 2001 ▶); data reduction: *SAINT-Plus*; program(s) used to solve structure: *SHELXS97* (Sheldrick, 2008 ▶); program(s) used to refine structure: *SHELXL97* (Sheldrick, 2008 ▶); molecular graphics: *PLATON* (Spek, 2009 ▶); software used to prepare material for publication: *SHELXL97*.

## Supplementary Material

Crystal structure: contains datablocks I, global. DOI: 10.1107/S1600536809042469/rk2154sup1.cif

Structure factors: contains datablocks I. DOI: 10.1107/S1600536809042469/rk2154Isup2.hkl

Additional supplementary materials:  crystallographic information; 3D view; checkCIF report

## Figures and Tables

**Table 1:** Hydrogen-bond geometry (Å, °)

*D*—H⋯*A*	*D*—H	H⋯*A*	*D*⋯*A*	*D*—H⋯*A*
N1—H1*D*⋯O2	0.863 (9)	2.052 (10)	2.8971 (17)	166.0 (19)
N4—H4*B*⋯N2^i^	0.865 (9)	2.342 (12)	3.160 (2)	157.9 (19)
N4—H4*A*⋯O1^ii^	0.868 (9)	2.216 (11)	3.061 (2)	164.2 (19)
N3—H3*D*⋯O1^iii^	0.857 (9)	2.018 (10)	2.8599 (17)	167.1 (19)
N2—H2*B*⋯O2^iv^	0.867 (10)	2.255 (13)	3.065 (2)	155 (2)
N2—H2*A*⋯O2^v^	0.863 (10)	2.400 (15)	3.152 (2)	145.7 (19)

Symmetry codes: (i) 
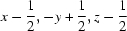
; (ii) 
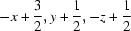
; (iii) 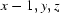; (iv) 
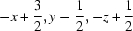
; (v) 
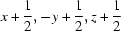
.

**Fig. 1.**
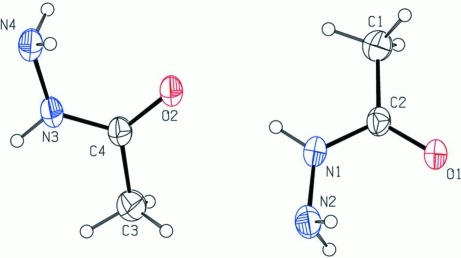
View of the asymmetric unit showing the atom–labelling scheme. Displacement ellipsoids are drawn at the 30% probability level. H atoms are presented as a small spheres of arbitrary radius.

## supplementary crystallographic information

### Comment

Hydrazide and its derivatives were used as versatile synthons. For example, substituted pyrazolones can be prepared by treatment of corresponding hydrazide with strong alkalies (Gagnon *et al.*, 1951). What's more, hydrazides are reactive functional groups routinely used in protein and carbohydrate chemistry (Raddatz *et al.*, 2002; Hermanson, 1996). It is reported that oligonucleotides can be modified with hydrazide (Lumley-Woodyear *et al.*, 1996). Acethydrazide is an important organic intermediate mainly for synthesis of nifuratrone in the pharmaceutical industry. Here we report the structure of the title compound (Fig. 1). Asymmetric unit contains two molecules with the same geometry. The crystal packing is stabilized by intermolecular classical N—H···O hydrogen bonds (Table 1).

### Experimental

Acethydrazide, prepared from ethyl acetate and 85% hydrazine was synthesized in 40% isolated yield. Crystals of acethydrazide suitable for X–ray data collection were obtained by cooled the reaction solution from 353 K to 293 K for overnight.

### Refinement

All H atoms of methyl groups were positioned geometrically with C—H = 0.96Å and *U*_iso_(H) = 1.5*U*_iso_(C). H atoms of amino–groups were found from the difference maps and refined with *U*_iso_(H) = 1.2*U*_iso_(N).

### Crystal data

**Table:**

C_2_H_6_N_2_O	*F*(000) = 320
*M**_r_* = 74.09	*D*_x_ = 1.205 Mg m^−^^3^
Monoclinic, *P*2_1_/*n*	Mo *K*α radiation, λ = 0.71073 Å
Hall symbol: -P 2yn	Cell parameters from 2153 reflections
*a* = 9.5636 (7) Å	θ = 2.5–28.0°
*b* = 8.7642 (6) Å	µ = 0.10 mm^−^^1^
*c* = 10.4282 (7) Å	*T* = 298 K
β = 110.886 (1)°	Block, colourless
*V* = 816.63 (10) Å^3^	0.20 × 0.15 × 0.10 mm
*Z* = 8	

### Data collection

**Table:**

Bruker SMART 4K CCD diffractometer	1762 independent reflections
Radiation source: fine-focus sealed tube	1604 reflections with *I* > 2σ(*I*)
graphite	*R*_int_ = 0.097
φ and ω scans	θ_max_ = 27.0°, θ_min_ = 2.5°
Absorption correction: multi-scan (*SADABS*; Bruker, 2001)	*h* = −10→12
*T*_min_ = 0.971, *T*_max_ = 0.990	*k* = −11→9
4189 measured reflections	*l* = −13→10

### Refinement

**Table:**

Refinement on *F*^2^	Secondary atom site location: difference Fourier map
Least-squares matrix: full	Hydrogen site location: inferred from neighbouring sites
*R*[*F*^2^ > 2σ(*F*^2^)] = 0.056	H atoms treated by a mixture of independent and constrained refinement
*wR*(*F*^2^) = 0.151	*w* = 1/[σ^2^(*F*_o_^2^) + (0.061*P*)^2^ + 0.1265*P*] where *P* = (*F*_o_^2^ + 2*F*_c_^2^)/3
*S* = 1.15	(Δ/σ)_max_ < 0.001
1762 reflections	Δρ_max_ = 0.19 e Å^−^^3^
112 parameters	Δρ_min_ = −0.18 e Å^−^^3^
6 restraints	Extinction correction: *SHELXL97* (Sheldrick, 2008), Fc^*^=kFc[1+0.001xFc^2^λ^3^/sin(2θ)]^-1/4^
Primary atom site location: structure-invariant direct methods	Extinction coefficient: 0.17 (2)

### Special details

**Table:**

Geometry. All s.u.'s (except the s.u. in the dihedral angle between two l.s. planes) are estimated using the full covariance matrix. The cell s.u.'s are taken into account individually in the estimation of s.u.'s in distances, angles and torsion angles; correlations between s.u.'s in cell parameters are only used when they are defined by crystal symmetry. An approximate (isotropic) treatment of cell s.u.'s is used for estimating s.u.'s involving l.s. planes.
Refinement. Refinement of *F*^2^ against ALL reflections. The weighted *R*–factor *wR* and goodness of fit *S* are based on *F*^2^, conventional *R*–factors *R* are based on *F*, with *F* set to zero for negative *F*^2^. The threshold expression of *F*^2^ > σ(*F*^2^) is used only for calculating *R*–factors(gt) *etc*. and is not relevant to the choice of reflections for refinement. *R*–factors based on *F*^2^ are statistically about twice as large as those based on *F*, and *R*–factors based on ALL data will be even larger.

### Fractional atomic coordinates and isotropic or equivalent isotropic displacement parameters (Å^2^)

**Table:**

	*x*	*y*	*z*	*U*_iso_*/*U*_eq_	
C1	0.8855 (2)	0.2274 (2)	0.0022 (2)	0.0608 (5)	
H1A	0.9481	0.3141	0.0057	0.091*	
H1B	0.7835	0.2603	−0.0227	0.091*	
H1C	0.8932	0.1562	−0.0649	0.091*	
C2	0.93450 (16)	0.15234 (18)	0.13942 (17)	0.0434 (4)	
C3	0.4604 (2)	0.0155 (2)	0.1911 (2)	0.0646 (5)	
H3A	0.4598	−0.0526	0.1188	0.097*	
H3B	0.3836	−0.0142	0.2254	0.097*	
H3C	0.5561	0.0111	0.2640	0.097*	
C4	0.43160 (16)	0.17510 (19)	0.13651 (16)	0.0443 (4)	
N1	0.83138 (14)	0.13848 (17)	0.19703 (16)	0.0501 (4)	
H1D	0.7430 (14)	0.176 (2)	0.1567 (19)	0.060*	
N2	0.86280 (16)	0.0740 (2)	0.32791 (17)	0.0575 (5)	
H2A	0.9337 (19)	0.128 (2)	0.3837 (19)	0.069*	
H2B	0.893 (2)	−0.0180 (14)	0.321 (2)	0.069*	
N3	0.30038 (14)	0.23464 (17)	0.12609 (15)	0.0490 (4)	
H3D	0.2363 (18)	0.182 (2)	0.148 (2)	0.059*	
N4	0.25433 (16)	0.38388 (19)	0.07867 (18)	0.0550 (4)	
H4B	0.257 (2)	0.398 (2)	−0.0025 (13)	0.066*	
H4A	0.3202 (19)	0.443 (2)	0.1362 (18)	0.066*	
O1	1.06315 (12)	0.10511 (15)	0.19701 (13)	0.0578 (4)	
O2	0.52482 (11)	0.24591 (14)	0.10257 (13)	0.0560 (4)	

### Atomic displacement parameters (Å^2^)

**Table:**

	*U*^11^	*U*^22^	*U*^33^	*U*^12^	*U*^13^	*U*^23^
C1	0.0487 (9)	0.0676 (12)	0.0619 (11)	−0.0009 (8)	0.0147 (8)	0.0087 (9)
C2	0.0331 (7)	0.0403 (8)	0.0566 (9)	−0.0026 (6)	0.0158 (6)	−0.0024 (7)
C3	0.0511 (10)	0.0578 (11)	0.0859 (14)	−0.0015 (9)	0.0256 (10)	0.0131 (10)
C4	0.0338 (7)	0.0516 (9)	0.0478 (8)	−0.0028 (6)	0.0149 (6)	−0.0019 (7)
N1	0.0330 (7)	0.0591 (9)	0.0590 (9)	0.0061 (6)	0.0174 (6)	0.0051 (7)
N2	0.0446 (8)	0.0717 (11)	0.0612 (10)	0.0017 (7)	0.0252 (7)	0.0019 (8)
N3	0.0350 (7)	0.0567 (9)	0.0605 (9)	−0.0027 (6)	0.0234 (6)	0.0018 (7)
N4	0.0385 (7)	0.0613 (10)	0.0690 (10)	0.0052 (6)	0.0237 (7)	0.0025 (8)
O1	0.0343 (6)	0.0723 (9)	0.0706 (8)	0.0085 (5)	0.0233 (6)	0.0194 (6)
O2	0.0369 (6)	0.0574 (7)	0.0806 (9)	0.0047 (5)	0.0292 (6)	0.0124 (6)

### Geometric parameters (Å, °)

**Table:**

C1—C2	1.491 (2)	C4—O2	1.2370 (18)
C1—H1A	0.9600	C4—N3	1.327 (2)
C1—H1B	0.9600	N1—N2	1.407 (2)
C1—H1C	0.9600	N1—H1D	0.863 (9)
C2—O1	1.2324 (18)	N2—H2A	0.863 (10)
C2—N1	1.331 (2)	N2—H2B	0.867 (10)
C3—C4	1.498 (3)	N3—N4	1.412 (2)
C3—H3A	0.9600	N3—H3D	0.857 (9)
C3—H3B	0.9600	N4—H4B	0.865 (9)
C3—H3C	0.9600	N4—H4A	0.868 (9)
			
C2—C1—H1A	109.5	O2—C4—N3	122.42 (16)
C2—C1—H1B	109.5	O2—C4—C3	121.57 (14)
H1A—C1—H1B	109.5	N3—C4—C3	116.01 (14)
C2—C1—H1C	109.5	C2—N1—N2	122.56 (13)
H1A—C1—H1C	109.5	C2—N1—H1D	120.0 (14)
H1B—C1—H1C	109.5	N2—N1—H1D	117.3 (14)
O1—C2—N1	121.40 (16)	N1—N2—H2A	106.0 (15)
O1—C2—C1	122.26 (15)	N1—N2—H2B	104.9 (16)
N1—C2—C1	116.34 (14)	H2A—N2—H2B	111 (2)
C4—C3—H3A	109.5	C4—N3—N4	124.09 (14)
C4—C3—H3B	109.5	C4—N3—H3D	120.8 (14)
H3A—C3—H3B	109.5	N4—N3—H3D	115.1 (14)
C4—C3—H3C	109.5	N3—N4—H4B	110.9 (14)
H3A—C3—H3C	109.5	N3—N4—H4A	104.5 (14)
H3B—C3—H3C	109.5	H4B—N4—H4A	109 (2)
			
O1—C2—N1—N2	2.0 (3)	O2—C4—N3—N4	−1.3 (3)
C1—C2—N1—N2	−178.17 (16)	C3—C4—N3—N4	179.13 (16)

### Hydrogen-bond geometry (Å, °)

**Table:**

*D*—H···*A*	*D*—H	H···*A*	*D*···*A*	*D*—H···*A*
N1—H1D···O2	0.86 (1)	2.05 (1)	2.8971 (17)	166 (2)
N4—H4B···N2^i^	0.87 (1)	2.34 (1)	3.160 (2)	158 (2)
N4—H4A···O1^ii^	0.87 (1)	2.22 (1)	3.061 (2)	164 (2)
N3—H3D···O1^iii^	0.86 (1)	2.02 (1)	2.8599 (17)	167 (2)
N2—H2B···O2^iv^	0.87 (1)	2.26 (1)	3.065 (2)	155 (2)
N2—H2A···O2^v^	0.86 (1)	2.40 (2)	3.152 (2)	146 (2)

Symmetry codes: (i) *x*−1/2, −*y*+1/2, *z*−1/2; (ii) −*x*+3/2, *y*+1/2, −*z*+1/2; (iii) *x*−1, *y*, *z*; (iv) −*x*+3/2, *y*−1/2, −*z*+1/2; (v) *x*+1/2, −*y*+1/2, *z*+1/2.
